# The alteration of left ventricular strain in later-onset spinal muscular atrophy children

**DOI:** 10.3389/fncel.2022.953620

**Published:** 2022-08-18

**Authors:** Yiqin Cui, Yijie Feng, Yu Xia, Xingpeng Fu, Ming Gong, Jingjing Qian, Jin Yu, Jingjing Ye, Feng Gao, Hongqiang Cheng, Shanshan Mao

**Affiliations:** ^1^Department of Neurology, National Clinical Research Center for Child Health, The Children’s Hospital, Zhejiang University School of Medicine, Hangzhou, China; ^2^Department of Ultrasound, National Clinical Research Center for Child Health, The Children’s Hospital, Zhejiang University School of Medicine, Hangzhou, China; ^3^Department of Pathology and Pathophysiology, Sir Run Run Shaw Hospital, Zhejiang University School of Medicine, Hangzhou, China

**Keywords:** spinal muscular atrophy, cardiovascular system, myocardial injury, left ventricular strain, serum lipid profile

## Abstract

**Background:**

Patients with spinal muscular atrophy (SMA) may suffer from multisystem injury, including an impaired cardiovascular system. However, M-mode echocardiography, the current dominant echocardiographic modality, is limited in the detection of myocardial injury. We considered the use of left ventricular strain imaging in detecting myocardial injury and explored the serum lipid profile related to cardiovascular disease in later-onset SMA children.

**Methods:**

A case-control study involving 80 patients with later-onset SMA and 80 age-, gender-, and body surface area-matched control children was conducted in a single tertiary pediatric hospital in China. Data on the left ventricular strain measured using two-dimensional speckle tracking echocardiography, left ventricular function parameters assessed by M-mode echocardiography, and serum lipid profile of these two groups were retrospectively collected for differential analysis.

**Results:**

The mean age of the 80 SMA patients were (6.87 ± 2.87) years, of which 46 were type 2 and 34 were type 3 patients. The global longitudinal strain (GLS) of the SMA group (−18.7 ± 2.9%, *p* < 0.001) was lower than that of the control group; the time to peak longitudinal strain (TTPLS) of the SMA group (22.9 ± 13.6 ms, *p* < 0.001) was higher than that of the control group, while left ventricular ejection fraction (LVEF) and left ventricular fractional shortening (LVFS), measured by the Teichholz method of M-mode echocardiography, showed no significant differences between the two groups. In addition, independent indicators for cardiovascular risk, including total cholesterol (TC)/HDL, low-density lipoprotein (LDL)/HDL, and Apo B/Apo A1 levels, were higher in SMA children than in the control group.

**Conclusion:**

Compared with healthy controls, later-onset SMA children presented with reduced GLS and prolonged TTPLS while the LVEF and LVFS values were within normal range. In particular, whether a reduced GLS or prolonged TTPLS in later-onset SMA compared to the control group can predict the risk of future cardiomyopathy remains to be investigated.

## Introduction

Spinal muscular atrophy (SMA) is a neuromuscular disease with autosomal recessive inheritance. Its pathological characteristics mainly manifest as the loss of lower motor neurons of the anterior horn of the spinal cord (Lunn and Wang, 2008), which is caused by a deficiency of the survival motor neuron (SMN) protein encoded by the *SMN1* gene, thus resulting in progressive proximal limb and trunk muscle weakness and atrophy (Wirth, 2000). The prevalence of SMA is approximately1/11,000 live births, and its carrier frequency is 1/40, making it one of the most common fatal and disabling neurodegenerative disorders in infancy (Sugarman et al., 2012). The clinical phenotypes of SMA in children can be classified into four subtypes according to the age of onset and achieved motor milestones: SMA type 0 (utero-onset SMA), type 1 (infantile-onset SMA), type 2 (intermediate SMA), and type 3 (mild SMA), with type 2 and 3 regarded as later-onset SMA (Mercuri et al., 2012, 2018; Finkel et al., 2018).

The deficiency of functional SMN proteins may cause multi-system impairment, along with a progressive decline in motor function (Hamilton and Gillingwater, 2013; Shababi et al., 2014; Faravelli et al., 2015; Simone et al., 2016; Finkel et al., 2018; Mercuri et al., 2018; Yeo and Darras, 2020). Furthermore, the presence of cardiac complications has been mentioned in the 2018 international consensus statement and clinical studies (Rudnik-Schöneborn et al., 2008; Wijngaarde et al., 2017; Finkel et al., 2018; Sokolska et al., 2020; Djordjevic et al., 2021b); cardiac structural abnormalities, mainly septal defects and abnormalities of the cardiac outflow tract, were predominantly reported in type 0 and 1 SMA, while cardiac rhythm disorders were most frequently observed in patients with type 2 and 3 SMA. However, long-term cardiac complications, such as myocardial infarction with cardiac hypertrophy, has been confirmed in later-onset SMA patients (Sokolska et al., 2020; Djordjevic et al., 2021b). To date, although no specific mechanism has been established to link cardiovascular injury with SMN protein deficiency, patients with SMA may be severely affected by these defects in the absence of effective cardiac monitoring.

In clinical practice, M-mode echocardiography is the primary imaging modality used to evaluate cardiac injuries. However, a limitation of M-mode echocardiography is that cardiac abnormalities are more likely to be detected with a certain degree of myocardial remodeling. Left ventricular (LV) strain can be used for the quantification of LV function (Smiseth et al., 2016). In addition, the global longitudinal strain (GLS) could be the best parameter for evaluating strain, which is more sensitive than LV ejection fraction (LVEF) for measuring systolic function, and may be applied to identify subclinical LV dysfunction in cardiomyopathies (Potter and Marwick, 2018). For instance, assessments of the early phases of cardiac involvement in Duchenne muscular dystrophy (DMD) patients revealed significantly reduced values of GLS in the patients’ group in the case of a normal LVEF (Amedro et al., 2019; Panovský et al., 2021). Similarly, we hypothesized that altered LV strain imaging values, as indicative of regional myocardial abnormalities, may be used to provide an early warning of myocardial injury in children with SMA; however, its applicability in the population of children with SMA is still worth investigating.

The main objective of the present study was to investigate the potential application of two-dimensional speckle tracking echocardiography (2D-STE) strain imaging in detecting myocardial injury in children with later-onset SMA and to observe the level of serum lipid profile that is related to the risk of cardiovascular diseases in SMA children.

## Materials and methods

### Study design and recruitment

We recruited a total of 80 children, aged 3–18 years, with SMA who were seen in the Department of Neurology at the Children’s Hospital of Zhejiang University School of Medicine in Hangzhou, China, for this case-control study. The study was conducted between October 2019 and March 2022. The inclusion criteria for this study were (1) an age of 3–18 years, (2) diagnosis of 5q SMA by genetic testing, (3) not receiving disease-modifying therapy or any other energy metabolism drugs at the time of observation, and (4) providing consent to be involved in the study. Exclusion criteria were (1) the presence of other comorbidities that may present with abnormal cardiac function and (2) an inability to cooperate with the completion of the ultrasound examination (Supplementary Figure 1). Age-, gender-, and body surface area (BSA)-matched healthy children were enrolled as controls. Recruited participants underwent a physical examination, and their medical history was thoroughly reviewed to collect demographic information and clinical characteristics. The study was approved by the Ethics Committee of the Children’s Hospital of Zhejiang University School of Medicine (2019-IRB-121), and informed consent was signed by the participants’ guardians.

### 2D-STE and M-mode assessment of left ventricular function

All participants underwent a thorough echocardiographic examination in the left lateral position using the EPIQ7 ultrasound system (Philips Medical Systems, Best, Netherlands). Standard apical four-chamber (AP4), two-chamber (AP2), three-chamber (AP3) views were acquired with a frame rate of 60–90 frames/s, and only images with at least three continuous measurable cardiac cycles were stored as raw data for off-line analysis. Strain is a dimensionless indicator of the alteration in length between two points. One-dimensional (1D) strain ε(t) is defined as follows:


$$\varepsilon \,\left(t\right)=\left(L\,\left(t\right)-L_{0}\right)/L_{0}$$

where *L*(*t*) is the segment length along the longitudinal direction at any time *t* in the cardiac cycle, and *L*_0_ is the initial length of the myocardium (Smiseth et al., 2016). For the off-line longitudinal strain analysis, the standard 2D-STE technique was performed manually by an experienced echocardiographer that tracked endocardial contours using a line method following the 18-segment model recommended by the American Society of Echocardiography. Strain images and mean GLS values were automatically obtained using Automated Cardiac Motion Quantification^A.I^ (aCMQ^A.I.^) software, which can calculate the peak systolic strain values as well as the time to peak strain. In addition, the M-mode LV images were collected and stored for off-line analysis for at least five cardiac cycles. LV dimensions and functional parameters, such as LVEF and LV fractional shortening (LVFS), were measured by the Teichholz method of M-mode echocardiography (M-Teich).

### Serum lipid profile measurement

Venous blood samples of all participants were collected in serum-separating tubes after an 8-h fast for biochemical analysis of serum lipid profile. Serum triglycerides (TG), total cholesterol (TC), apolipoprotein A1 (Apo A1), apolipoprotein B (Apo B), high density lipoprotein (HDL), and low-density (LDL) lipoprotein cholesterol were measured using an automatic biochemical analyzer (Beckman Coulter Inc., United States). In addition, TC/HDL, LDL/HDL, and Apo B/Apo A1 ratios, as independent indicators, especially for evaluating cardiovascular disease risk (Millán et al., 2009), were also included.

### Statistical analysis

Normally distributed quantitative data are presented as mean ± standard deviation (SD). A paired *t*-test was utilized for analyzing differences between groups of normally distributed data, and non-normally distributed data were analyzed using the Steel-Dwass test. The Pearson’s linear correlation coefficient *r* was calculated to evaluate the correlations between lipid metabolism and GLS in SMA. All statistical analyses were performed using SPSS Statistics (version 25.0, IBM Corporation, Armonk, New York, United States). A *p*-value < 0.05 was considered statistically significant.

## Results

### Clinical characteristics of the patients and control children

Children with type 2 and 3 SMA (*n* = 80) and healthy age-, gender-, and BSA-matched control children (*n* = 80) were involved in this study. There were 46 (57.5%) patients with SMA type 2 and 34 (42.5%) with SMA type 3. No significant differences were observed between the general profile of patients with SMA and those of the controls (Table 1).

**TABLE 1:** Demographic and anthropometric data of SMA and control groups.

Clinical characteristic	Control	SMA	*p*-value
**Gender, *n* (%)**			
Male	41 (51.3%)	41 (51.3%)	–
Female	39 (48.7%)	39 (48.7%)	–
SMA type, *n* (%)	–		
SMA II	–	46 (57.5%)	–
SMA III	–	34 (42.5%)	–
Age years, mean ± SD	6.84 ± 2.88	6.85 ± 2.87	0.670
3∼6 years	38 (47.5%)	38 (47.5%)	
6∼9 years	27 (33.8%)	28 (35.0%)	
9∼12 years	8 (10.0%)	7 (8.8%)	
≥12 years	7 (8.8%)	7 (8.8%)	
Height, cm, mean ± SD	115.36 ± 19.28	115.38 ± 19.36	0.607
Weight, kg, mean ± SD	21.21 ± 8.63	21.21 ± 8.64	0.516
Body surface area, m^2^, mean ± SD	0.82 ± 0.22	0.82 ± 0.22	0.567
Body mass index, kg/cm^2^, mean ± SD	15.66 ± 3.92	15.65 ± 3.91	0.484

### Left ventricular strain and M-mode assessment of left ventricular functional parameters

**FIGURE 1:**
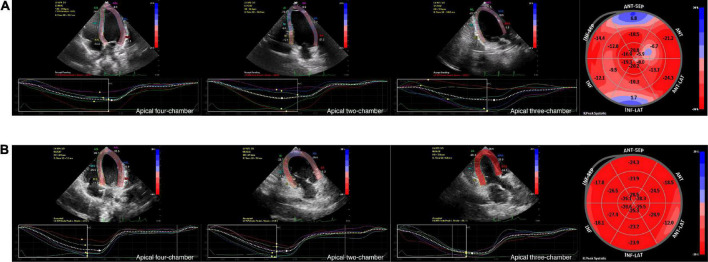
Graphic example of 2D-STE analyses to derive LV longitudinal strain. Measurement of LV longitudinal strain images in AP4, AP2 and AP3 view, and segmental Bull’s eye plot (from left to right) of an 8-year-old boy with SMA **(A)** and an age-, gender, BSA-matched healthy control **(B)** by 2D-STE. Bull’s eye plot is color-coded by strain deformation, with red color regions showing longitudinal strain within normal values, pale pink color regions showing negative displacement of strain, and blue color regions showing positive displacement of strain to varying degrees.

LV strain, as assessed by 2D-STE, and LV dimensions, LVEF, and LVFS, as measured by M-Teich, in SMA patients and controls were examined. Significant differences between the two groups were also observed in longitudinal strain and TTPLS under different views, such as AP4, AP2, and AP3. The GLS was significantly decreased in SMA children (−18.7 ± 2.9%, *p* < 0.001) compared to healthy controls (−23.3 ± 1.9%) (Figure 1). The TTPLS was significantly prolonged in SMA children compared to healthy controls (22.9 ± 13.6 ms and 14.2 ± 9.2 ms, respectively; *p* < 0.001). However, no significant differences were observed in LV dimensions measured by M-Teich between SMA children and controls. Regarding LV function parameters analyzed by M-Teich, a difference in LVEF was found between the two groups, but it was within the normal range of the reference value (LVEF ≥ 50%), while there was no significant difference in LVFS between the two groups (Table 2).

**TABLE 2:** 2D-STE assessment of LV strain and time to peak strain and M-mode assessment of LV function parameters of SMA and control groups.

Characteristic	Control (*n* = 80)	SMA (*n* = 80)	*p*-value
	Mean ± SD	Mean ± SD	
**2D-STE**			
**Longitudinal strain, %**			
Global^#^	−23.3 ± 1.9	−18.7 ± 2.9	**<0.001**
Apical four-chamber	−23.7 ± 2.2	−18.9 ± 3.9	**<0.001**
Apical two-chamber	−23.3 ± 2.5	−18.6 ± 2.9	**<0.001**
Apical three-chamber	−23.0 ± 1.9	−18.1 ± 2.9	**<0.001**
**Time to peak longitudinal strain, %**			
Global^#^	14.2 ± 9.2	22.9 ± 13.6	**<0.001**
Apical four-chamber	28.3 ± 8.2	35.6 ± 14.9	**<0.001**
Apical two-chamber	29.5 ± 6.6	37.6 ± 16.7	**<0.001**
Apical three-chamber	28.8 ± 10.8	44.5 ± 28.4	**<0.001**
**M-mode LV functional parameters**			
IVSs, cm	0.92 ± 0.24	0.89 ± 0.19	0.424
LVDd, cm	3.21 ± 0.80	3.16 ± 0.61	0.690
LVDs, cm	2.50 ± 0.53	2.45 ± 0.53	0.002
LVPWDd, cm	0.70 ± 0.14	0.68 ± 0.37	0.669
LVPWDs, cm	0.84 ± 0.19	0.85 ± 0.21	0.762
LVEF, %	69.77 ± 4.45	68.32 ± 3.59	0.047
LVFS, %	37.42 ± 3.71	38.45 ± 4.55	0.077

^#^Average of longitudinal strain of four-, two-, and three-chamber views. *IVSs*, intraventricular septum dimension in systole; LVDd, *l*eft ventricle internal dimension in diastole; LVDs, left ventricle internal dimension in systole; LVPWDd, *l*eft ventricle posterior wall dimension in diastole; LVPWDs, left ventricle posterior wall dimension in systole. A *p*-value < 0.05 is considered statistically significant (bold font).

### Serum lipid profile measurement

There were differences in the serum lipid profile between the two groups. The HDL and Apo Al levels were decreased while TG was elevated in the SMA group, compared with those in the control group. Independent predictors of the lipoprotein ratio, including TC/HDL, LDL/HDL, and Apo B/Apo A1, were elevated in the SMA group (Table 3).

**TABLE 3:** Serum lipid profile levels of SMA and control groups.

Serum lipid profile	Control (*n* = 80)	SMA (*n* = 80)	*p*-value
TG, mmol/L, mean ± SD	0.82 ± 0.38	1.01 ± 0.48	**0.004**
TC, mmol/L, mean ± SD	4.42 ± 1.23	4.54 ± 0.78	0.398
HDL, mmol/L, mean ± SD	1.38 ± 0.23	1.14 ± 0.30	**<0.001**
LDL, mmol/L, mean ± SD	2.65 ± 0.62	2.75 ± 0.51	0.158
Apo Al, mmol/L, mean ± SD	1.40 ± 0.13	1.23 ± 0.24	**<0.001**
Apo B, mmol/L, mean ± SD	0.75 ± 0.26	0.80 ± 0.18	0.107
TC/HDL, mean ± SD	3.27 ± 1.10	4.14 ± 1.13	**<0.001**
LDL/HDL, mean ± SD	1.97 ± 0.54	2.52 ± 0.66	**<0.001**
Apo B/Apo A1, mean ± SD	0.54 ± 0.19	0.68 ± 0.26	**<0.001**

TG, triglycerides; TC, total cholesterol; HDL, high density lipoprotein cholesterol; LDL, low-density lipoprotein cholesterol; Apo B, apolipoprotein B; Apo A1, apolipoprotein A1. A *p*-value < 0.05 is considered statistically significant (bold font).

### Correlations of global longitudinal strain, time to peak longitudinal strain, and serum lipid profile in spinal muscular atrophy

To further investigate whether abnormal serum lipid profile in children with SMA may be related to myocardial injury, a correlation analysis was conducted. As shown in Table 4, the TC/HDL, LDL/HDL, and Apo B/A1 ratios (Pearson *r* = 0.308, *p* = 0.006; *r* = 0.574, *p* < 0.001; and *r* = 0.228, *p* = 0.042, respectively), as well as HDL levels (*r* = −0.380, *p* = 0.001) were also correlated with LV GLS. No correlation was observed between lipoproteins and LV TTPLS. Additionally, general demographic indicators, such as the age, BSA, and body mass index (BMI) (*r* = 0.499, *p* < 0.001; *r* = 0.504, *p* < 0.001; and *r* = 0.364; *p* = 0.001, respectively) were correlated with LV GLS in patients with type 2 and 3 SMA, which was also observed in the control group (data not shown).

**TABLE 4:** Pearson correlation coefficient of LV global longitudinal strain and time to peak longitudinal strain of the SMA group.

Independent variable	Global longitudinal strain		Time to peak longitudinal strain	
	Pearson’s r	*p*-value	Pearson’s r	*p*-value
Age	0.499	**<0.001**	0.255	**0.022**
BSA	0.504	**<0.001**	0.157	0.165
BMI	0.364	**0.001**	−0.182	0.106
TG	0.120	0.287	0.092	0.419
TC	0.049	0.664	0.172	0.128
TC/HDL	0.308	**0.006**	x -0.260	**0.020**
HDL	−0.380	**0.001**	0.146	0.197
LDL	0.176	0.118	0.048	0.672
LDL/HDL	0.574	**<0.001**	−0.129	0.255
Apo A1	−0.196	0.081	−0.031	0.781
Apo B	0.103	0.362	−0.055	0.628
Apo B/Apo A1	0.228	**0.042**	−0.040	0.724

BSA, body surface area; BMI, body mass index; TG, triglycerides; TC, total cholesterol; HDL, high density lipoprotein cholesterol; LDL, low-density lipoprotein cholesterol; Apo B, apolipoprotein B; Apo A1, apolipoprotein A1. A *p*-value < 0.05 is considered statistically significant (bold font).

## Discussion

In this study, we collected 2D-STE strain imaging values, data from M-mode echocardiographic measurements, and serum lipid profile of type 2 and 3 SMA patients and age-, gender- and BSA-matched healthy control children; to our knowledge, this is the first study investigating LV strain values in the SMA population to detect potential myocardial injury. A significant decrease in LV GLS and prolonged TTPLS detected by 2D-STE strain imaging, no significant alternations in LV functional parameters measured by M-Teich, and abnormal serum lipid profile was observed in SMA patients. In addition, greater significant LV dysfunction, as indicated by the longitudinal strain, was observed in SMA patients with a LDL/HDL at higher risk levels.

LV strain, a means to quantify LV function, is feasible with 2D-STE. Recently, studies have increasingly used LV strain to assess regional myocardial deformations, which can directly reflect the regional myocardial function, independent of peripheral segments. GLS, a key indicator of LV strain, is widely used in the study of various diseases that may be complicated by subclinical cardiac impairment, such as hypertension (Tadic and Cuspidi, 2021a), heart transplantation (Godown et al., 2018), and type 2 diabetes (Tadic and Cuspidi, 2021b). Panovský et al. (2021) conducted a case-control study in children with DMD using cardiac magnetic resonance strain analysis for the detection of early LV dysfunction, and a decreased global LV strain was observed, even in the case of a normal LVEF, providing evidence for the use of LV strain in neuromuscular disease. The presence of cardiac manifestations in children with SMA has been reported in several clinical studies (Wijngaarde et al., 2017), and great importance has been attached to severe SMA type 0 and 1 patients with cardiac structural abnormalities from an early stage of the disease (Wijngaarde et al., 2017). Patients with type 2 and 3 SMA, however, are less likely to manifest with significant clinical, electrocardiographic, or echocardiographic signs of cardiomyopathy; thus, they do not often undergo regular cardiac monitoring. Nevertheless, cases have been reported in which serious complications such as myocardial infarction with cardiac hypertrophy may still occur with aging (Sokolska et al., 2020; Djordjevic et al., 2021b). Cardiac abnormalities and autonomic defects have also been reported in SMA mouse models (Bevan et al., 2010; Shababi et al., 2010; Sheng et al., 2018). For example, G0/G1 cell cycle arrest and cell proliferation defect were found in SMA mouse cardiac myocytes due to the lack of SMN protein, and the ensuing disturbance of cardiac blood flow would further affect myocardial tissue growth factor expression, thus interfering with normal ventricular development (Sheng et al., 2018). Compared with controls, SMA mice with a milder phenotype had no significant alterations in cardiac function at birth, but pathological changes of myocardial injury, such as myocardial contractile protein rearrangement, myocardial cell disorder, and myocardial interstitial fibrosis, gradually appeared (Bevan et al., 2010; Sheng et al., 2018). Hence, applying more sensitive observations to the early detection of potential myocardial injury is essential, especially for patients with later-onset SMA.

In this study, we adopted the technique of 2D-STE strain imaging to detect potential cardiac impairment in children with SMA by conducting a case-control study and reached a result similar to Panovský et al. (2021) study, wherein GLS was significantly decreased in patients compared with that in controls, while no significant difference was found in LVEF of patients and controls. LV strain findings further described its capacity for the accurate determination of regional myocardial motion and can be used for an early warning of potential myocardial injury.

As previously reported, Levy et al. (2016) defined reference values of 2D-STE derived LV strain in healthy children based on 43 systematic valid data sets, and the mean GLS value was −20.2%. Our results for children with type 2 and 3 SMA demonstrated significantly lower GLS than those in healthy children, while the GLS of controls in our study was defined as normal, further providing evidence of abnormal cardiac conditions in SMA, using LV strain imaging. Therefore, our study supports the evidence of clinically recognized early myocardial changes in later-onset SMA patients using LV strain. Notably, M-Teich measured within-normal-range values of LVEF and LVFS in SMA patients in this study.

Our study further examined the serum lipid profile of children with SMA, which is emphasized in cardiovascular disorders (Mansell et al., 2022). The consistent finding with previous studies in SMA patients indicates the role of SMN protein depletion in abnormal serum lipid profile, mainly focusing on glucose and fatty acid metabolism abnormalities (Deguise et al., 2019; Djordjevic et al., 2021a). The progressive muscle weakness and atrophy caused by disease leads to significantly reduced daily activities, further leading to increased fat mass storage, relatively low caloric consumption and eventually an altered body composition. Genetically related mechanisms of abnormal lipid metabolism are also proposed such as defects in neighboring genes, or the loss of a neural “trophic factor” (Li et al., 2020). Thus, in patients with later-onset SMA, obesity-related changes in serum lipid profile may be observed. In this study, HDL and Apo A1 levels, which are negatively associated with cardiovascular disease risk, were significantly decreased in SMA children, while the cardiovascular risk indicators, TC/HDL, LDL/HDL, and Apo B/Apo A1 ratios (Millán et al., 2009), were significantly higher, suggesting a potentially increased risk of cardiovascular diseases. Additionally, the correlation analysis between serum lipid profile and LV strain suggested that LDL/HDL was correlated with LV strain, a preliminary indication that abnormal levels of serum lipid profile may be one of the factors influencing alterations of myocardial strain indicators in children with SMA; thus, further research is required.

This study is the first to examine LV strain values in children with later-onset SMA and will help further define early predictors of myocardial injury in SMA patients. We analyzed the use of LV strain measured using 2D-STE imaging in children with SMA and explored the correlation between specific LV strain and serum lipid profile values. In addition, this study had several limitations. First, the majority of study participants were children aged 4–9 years; therefore the study may not be able to fully cover the entire range of SMA patients, especially adolescents. Second, because of the traditional observational case-control study design, changes in LV function in these children over the course of the disease and the effects of drug therapy still need to be followed longitudinally.

## Conclusion

In conclusion, children with SMA have altered LV strain values compared with healthy controls, as evidenced by a reduced GLS, which may have the potential of being a sensitive indicator for the early detection of myocardial injury in later-onset SMA children, and abnormal lipid levels in children with SMA may be related with a reduced GLS. Whether reduced GLS and prolonged global TTPLS values in SMA can predict the risk of cardiomyopathy remains to be investigated.

## Data availability statement

The raw data supporting the conclusions of this article will be made available by the authors, without undue reservation.

## Ethics statement

The studies involving human participants were reviewed and approved by the Ethics Committee of the Children’s Hospital of Zhejiang University School of Medicine (2019-IRB-121). Written informed consent to participate in this study was provided by the participants’ legal guardian/next of kin.

## Author contributions

YC, YF, and YX performed the primary data analysis and wrote the manuscript. XF, MG, JQ, JY, and JJY collected the clinical data. YC and YX performed the statistical analysis of the data. FG supported and helped revise the study. HC assisted in supervising the completion of the study. SM designed and supervised the study. All authors contributed to the article and approved the submitted version.

## Supplementary material

Supplementary Figure 1 Flow chart of patient inclusion and exclusion.
