# Corrigendum: The alteration of left ventricular strain in later-onset spinal muscular atrophy children

**DOI:** 10.3389/fncel.2022.1028583

**Published:** 2022-09-30

**Authors:** Yiqin Cui, Yijie Feng, Yu Xia, Xingpeng Fu, Ming Gong, Jingjing Qian, Jin Yu, Jingjing Ye, Feng Gao, Hongqiang Cheng, Shanshan Mao

**Affiliations:** ^1^Department of Neurology, National Clinical Research Center for Child Health, The Children's Hospital, Zhejiang University School of Medicine, Hangzhou, China; ^2^Department of Ultrasound, National Clinical Research Center for Child Health, The Children's Hospital, Zhejiang University School of Medicine, Hangzhou, China; ^3^Department of Pathology and Pathophysiology, Sir Run Run Shaw Hospital, Zhejiang University School of Medicine, Hangzhou, China

**Keywords:** spinal muscular atrophy, cardiovascular system, myocardial injury, left ventricular strain, serum lipid profile

In the published article, there was an error in Results, Left ventricular strain and M-mode assessment of left ventricular functional parameters, Paragraph 1. The results of global longitudinal strain (GLS) in SMA children and healthy controls were incorrectly stated. The results were previously stated as “The GLS was significantly decreased in SMA children (−23.3 ± 1.9%, *p* < 0.001) compared to healthy controls (−18.7 ± 2.9%)” but should be, “The GLS was significantly decreased in SMA children (−18.7 ± 2.9%, *p* < 0.001) compared to healthy controls (−23.3 ± 1.9%).” The corrected paragraph appears below:

LV strain, as assessed by 2D-STE, and LV dimensions, LVEF, and LVFS, as measured by M-Teich, in SMA patients and controls were examined. Significant differences between the two groups were also observed in longitudinal strain and TTPLS under different views, such as AP4, AP2, and AP3. The GLS was significantly decreased in SMA children (−18.7 ± 2.9%, *p* < 0.001) compared to healthy controls (−23.3 ± 1.9%) (**Figure 1**). The TTPLS was significantly prolonged in SMA children compared to healthy controls (22.9 ± 13.6 ms and 14.2 ± 9.2 ms, respectively; *p* < 0.001). However, no significant differences were observed in LV dimensions measured by M-Teich between SMA children and controls. Regarding LV function parameters analyzed by M-Teich, a difference in LVEF was found between the two groups, but it was within the normal range of the reference value (LVEF ≥ 50%), while there was no significant difference in LVFS between the two groups (**Table 2**).

The authors apologize for this error and state that this does not change the scientific conclusions of the article in any way. The original article has been updated.

## Publisher's note

All claims expressed in this article are solely those of the authors and do not necessarily represent those of their affiliated organizations, or those of the publisher, the editors and the reviewers. Any product that may be evaluated in this article, or claim that may be made by its manufacturer, is not guaranteed or endorsed by the publisher.

