# Supplementary figures and images for: The alteration of left ventricular strain in later-onset spinal muscular atrophy children
Source: Front Cell Neurosci. 2022 Aug 18;16:953620. doi: 10.3389/fncel.2022.953620 (PMC9435971; doi:10.3389/fncel.2022.953620)

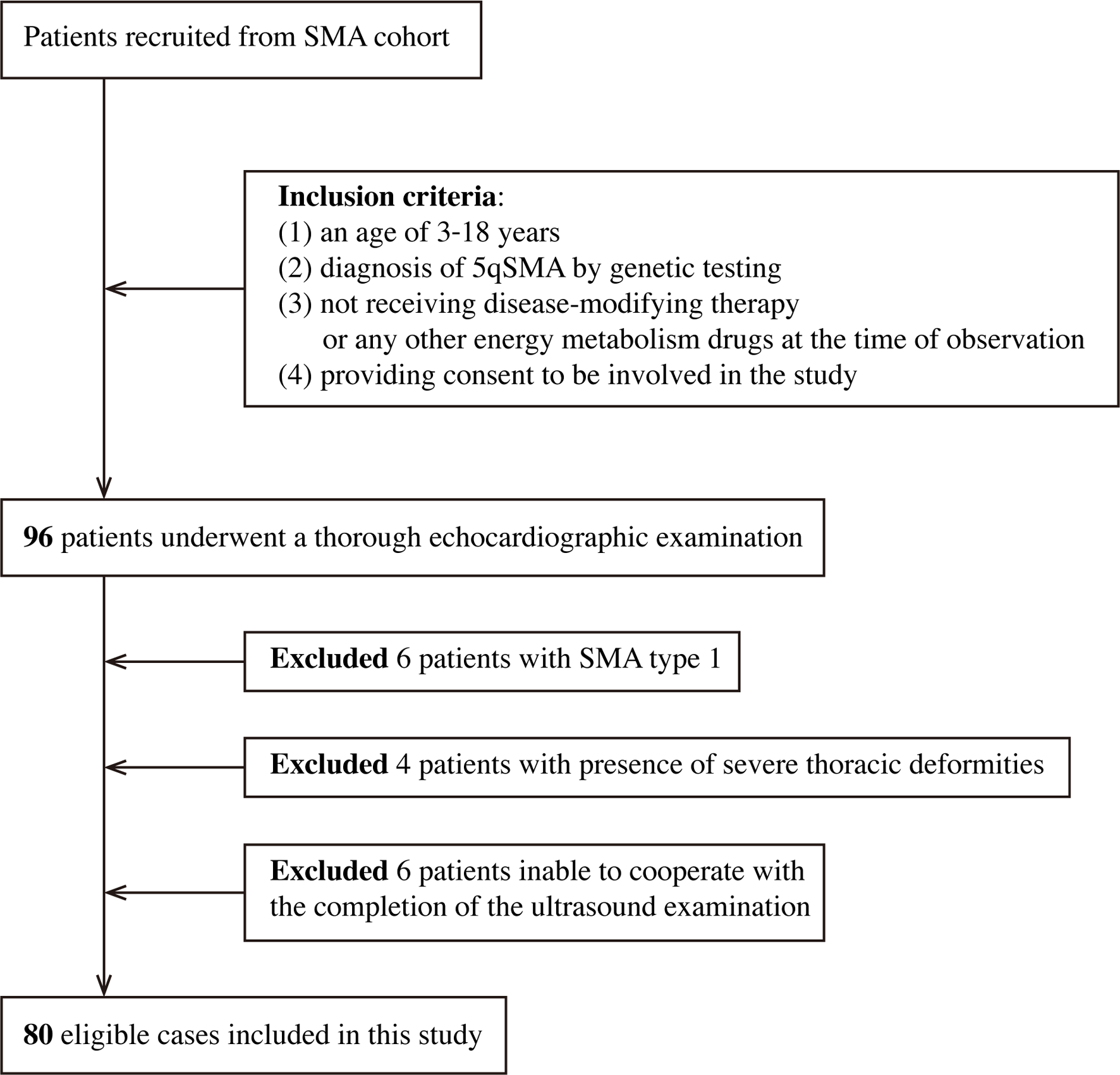

Supplement: Supplementary Figure 1 — Flow chart of patient inclusion and exclusion. [file Image_1.tif]
